# Using natural language processing techniques to inform research on nanotechnology

**DOI:** 10.3762/bjnano.6.149

**Published:** 2015-07-01

**Authors:** Nastassja A Lewinski, Bridget T McInnes

**Affiliations:** 1 Department of Chemical and Life Science Engineering, Virginia Commonwealth University, Richmond, VA, USA; 2 Department of Computer Science, Virginia Commonwealth University, Richmond, VA, USA

**Keywords:** data mining, informatics, name entity recognition, nano-informatics, nanoparticles, nanotechnology, nanotoxicity, natural language processing, text mining

## Abstract

Literature in the field of nanotechnology is exponentially increasing with more and more engineered nanomaterials being created, characterized, and tested for performance and safety. With the deluge of published data, there is a need for natural language processing approaches to semi-automate the cataloguing of engineered nanomaterials and their associated physico-chemical properties, performance, exposure scenarios, and biological effects. In this paper, we review the different informatics methods that have been applied to patent mining, nanomaterial/device characterization, nanomedicine, and environmental risk assessment. Nine natural language processing (NLP)-based tools were identified: NanoPort, NanoMapper, TechPerceptor, a Text Mining Framework, a Nanodevice Analyzer, a Clinical Trial Document Classifier, Nanotoxicity Searcher, NanoSifter, and NEIMiner. We conclude with recommendations for sharing NLP-related tools through online repositories to broaden participation in nanoinformatics.

## Introduction

Nanotechnology may still be considered a relatively new field. However, its impact is already realized with engineered nanomaterials (ENMs) incorporated in over 1800 consumer products, included in over 100 clinical trials, and contained in 40 FDA approved nanomedicines [1–3]. At the onset of the U.S. National Nanotechnology Initiative, researchers spearheaded efforts to “get it right the first time” by studying the potential human health and environmental impacts of ENMs in parallel with ENMs discovery and development. However, the creation and establishment of data repositories as well as algorithms to automatically analyze the collected resources has lagged behind. As a consequence, unlike bioinformatic areas such as genomics or systems biology, nanoinformatics is still in its infancy.

Nanoinformatics is defined as “the science and practice of determining which information is relevant to the nanoscale science and engineering community, and then developing and implementing effective mechanisms for collecting, validating, storing, sharing, analyzing, modeling, and applying that information” [4]. Applications of nanoinformatics include data integration and exchange (e.g., caNanoLab, GoodNanoGuide), nanoparticle characterization (e.g., caNanoLab, Nanomaterial Registry), domain ontologies (e.g., NanoParticle Ontology), terminologies and standards (e.g., ISA-TAB-Nano), data and text mining (e.g., NEIminer, TechPerceptor), and modeling/simulation (e.g., HDAT). Extracting information usually comes from two different sources: (1) literature to which natural language processing methods are applied, and (2) experimental data to which data modeling methods, such as those used in HDAT and NanoMiner, are applied [5–6]. Despite being a largely overlooked area of informatics, several reviews have been published that list the different databases and tools currently available [7–11]. In this review, we focus on the tools that utilize natural language processing.

Natural language processing (NLP) involves the use of computers to perform practical tasks involving written language, such as extracting and analyzing information from unstructured text. What separates NLP applications from other data processing systems is their use of knowledge about human language [12]. Many of the NLP applications utilize literature retrieved from databases. Information retrieval, document classification, and pattern matching methods are often utilized to ensure that the documents being analyzed by the NLP systems contain relevant engineered nanomaterials information [13–14].

In the nanoinformatics literature discussed in this review, there are several NLP methods and systems that were proposed to extract, classify, and understand ENM-related information within unstructured text. One of the most commonly explored NLP applications by nanoinformatics researchers was Entity Extraction, which is the task of identifying mentions of a specific entity within unstructured text. The entities explored by nanoinformatics researchers varied between very specific entities such as the particle diameter of a poly(amidoamine) dendrimer [15] to very broad such as any toxicological hazard of nanoparticles [16]. Within the literature, there was also a discussion of the prospective NLP tools and algorithms that may be useful to provide information about a set of nanotechnology related documents. For example, the development of a topic identification and summarization component was proposed for incorporation into the NanoPort system to provide researchers with an automatically generated abstract or listing of relevant information based on a document [13].

Terminologies and taxonomies are equally important when building many of the NLP-based algorithms. Information Retrieval and Entity Extraction can be guided by relevant ontologies. Thomas et al. developed the first NanoParticle Ontology (NPO) based on the Open Biomedical Ontologies (OBO) Foundry principles, which were set up to promote the standardization of ontologies and common controlled vocabularies for data integration [17–18]. Recently, the eNanoMapper project has developed an ontology that merges and extends existing ontologies, including the NPO [19]. Ontologies in other languages, such as Japanese and Russian, have also been developed [20–21]. In the following section, we describe our method for identifying the nanoinformatics literature discussed in this paper, and then review the different informatics methods that have been applied such as patent mining, nanomaterial/device characterization, nanomedicine, and environmental risk assessment.

## Methods

This review was limited to the English language literature included in two databases, PubMED and Web of Science [22–23]. The searches were conducted on February 12, 2015. For the search term (nano* AND “natural language processing”), Web of Science retrieved 5 records (2 excluded) and PubMED retrieved 2 records (2 excluded). For the search term (nanoinformatic*) Web of Science retrieved 38 records (34 excluded) and PubMED retrieved 24 records (22 excluded). For the search term (nano* AND “text mining”), Web of Science retrieved 38 records (34 excluded) and PubMED retrieved 2 records (2 excluded).

The following exclusion criteria were applied to the retrieved records:

- Bioinformatics papers not specifically focused on nanotechnology were not included.
- Bibliometric approaches were not included.
- Non-text based approaches (such as QSAR or image analysis) were not included.
- NLP approach(es) not described in full detail were not included.

After excluding duplicates, an initial set of 7 papers was retrieved using the described Boolean searches. We then expanded our search to include the literature cited within these 7 papers as well as the literature citing these 7 papers as identified in PubMED and Web of Science. A final set of 14 papers were included for detailed review, and the results are presented in the following section.

## Review

### Patent mining

Three groups across the globe (USA, Japan, China) have developed independent, NLP-based patent text mining systems. NLP is not the only approach to text mining and we refer the reader to a recent review by Abbas et al. on the state of the art in patent analysis [24].

#### NanoPort

NanoPort is a web portal that (1) automatically identifies nano-related documents (website articles, patent documents, and academic articles), and (2) supports the searching and analysis of the documents [13]. The portal contains a content analysis module that utilizes NLP technology in order to help the researcher to understand and analyze the documents returned by the search engine of the portal. The authors proposed to include (1) a document summarizer, (2) a document clusterer, (3) a topic mapper, and (4) a patent analyzer.

The proposed document summarizer automatically develops an abstract containing the important points of the document for the researcher. The authors propose using their previously developed Arizona Txttractor system, which was initially developed for web pages. The document clusterer groups the documents returned by the portal based on common topics identified within the document using the author’s Arizona Noun Phraser (ANP). ANP identifies noun phrases in text and then ranks them based on their frequency. The highly frequent noun phrases are used as topics by the clusterer as well as to support visualization of the search results in the topic mapper. The proposed Patent analyzer supports the basic analysis, content map analysis and citation network analysis. The basic analysis contains traditional patent analysis information such as number of patents based on country, institution or technology field. The content map allows for the concepts from multiple patents to be viewed and analyzed over time. The patent citation network allows for the visualization of links between entities such as countries, institutions and technology fields providing a wider scope of the field for the researcher. NanoPort was hosted at http://www.nanoport.org but unfortunately is no longer available online.

#### NanoMapper

NanoMapper expands on the proposed patent analyzer within the NanoPort system [25]. The NanoMapper prototype provides search capability, visualization and analytical tools to analyze nanotechnology patents from the United States Patent and Trademark Office (USPTO), European Patent Office (EPO), Japan Patent Office (JPO), and grants from the U.S. National Science Foundation (NSF). It includes basic statistics, citation network analysis and content map analysis as described in the proposed NanoPort patent analyzer as well as publication trend analysis to compare trends of patents and grants. Similarly to NanoPort, the NSF-funded NanoMapper was hosted at http://nanomapper.eller.arizona.edu but is no longer available online.

#### TechPerceptor

TechPerceptor is a text mining tool to conduct patent analysis and generate a patent map based on a subject–action–object (SAO) approach [26–28]. Their training corpus consisted of 136 patents and was initially analyzed for trends in carbon nanotube synthesis methods [26–27]. More recently, the research group expanded the scope to include applications of carbon nanotubes such as incorporation in photovoltaic cells and prostate cancer therapeutics [28]. The patents, which spanned the years 1992 to 2009, were collected from E.U., Japan, Korea and U.S. patent databases with patents in Japanese and Korean translated using K2E-PAT or Google Translate. The group followed a four step procedure for both their SAO-based static and dynamic patent map construction: 1) collect patent data, 2) extract SAO structures using NLP, (3) generate a patent dissimilarity matrix, and (4) visualize as dynamic patent [26–27]. The patent maps were also automatically analyzed to identify areas of high or low activity, infringement and novelty, which were determined based on degrees of (dis)similarity to other patents [28].

Their static tool revealed 8 patent clusters with the most patents reporting arc-discharge and laser vaporization synthesis methods [26]. Chemical vapor deposition (CVD) methods were also mentioned as being invented frequently. Top patenting companies included NEC, Samsung and Sony. Their dynamic tool revealed a possible patent vacuum of using low temperature or microwave-based synthesis of single-walled carbon nanotubes [27]. Analyzing hot spots revealed changes in the type of synthesis method patented over time, with synthesis methods evolving from arc discharging in 1999–2000 to metal-catalyzed heat-treatment syntheses and CVD in 2003–2004, to arc discharge with purification control in 2005–2006, to plasma-enhanced and thermal CVD in 2007–2010. CVD is the dominant commercial synthesis approach and catalyzed CVD with fluidized bed has been used by Bayer to synthesize Baytubes [29]. Competitor analysis revealed overlap between Sony and an individual researcher, Young Sang Cho.

#### Text mining framework for Nano S&T

Junpeng et al. developed a patent text mining tool using NLP [14]. Patents were retrieved from Science Citation Index, Engineering Information Compendex, International Information Services for Physics and Engineering communities, and the Chinese Patent database. Text extraction was conducted, with fuzzy logic used to cleanse the data. Fuzzy matching techniques were used to identify and combine similar entities. List Process, Matrix Process, Factor Analysis, Technology Group Clustering, and Concept Hierarchy were used in the framework to analyze the database. Multi-dimensional scaling was employed with a path erasing algorithm. The data presented focused on identifying leading countries, companies and inventors in the nanotechnology field. At the time of publication, the top three patenting institutions representing the top three patenting countries included the Naval Research Laboratory (USA), Cavendish Laboratory (UK), and Hitachi Ltd (Japan).

### Nanomaterial/device characterization

Not all ENMs or nanodevices and their respective synthesis or fabrication methods are patented. In addition, the information provided in a patent can be limited compared to that included in a research article. Therefore systems that can automatically retrieve and annotate literature on ENMs/nanodevices can be valuable tools for accelerating the discovery/design, synthesis/fabrication and optimization of ENMs/nanodevices.

#### Nanodevice fabrication and characterization analyzer

Dieb et al. generated a tool to automatically collect literature relevant to nanodevice design and a tool to automatically annotate literature on nanodevices [30–31]. A training set, which consisted of two fully annotated papers with 129 sentences, was manually annotated by graduate students with the assistance of an annotation support tool, XConc Suite [32]. The terms included: source material (SMaterial), characteristic feature of material (SMChar), experiment parameter (ExP), value of the experiment parameter (ExPVal), evaluation parameter (EvP), value of the evaluation parameter (EvPVal), manufacturing method (MMethod), and final product (TArtifact).

Because terms can overlap with other terms, four tag groups were created where the terms within a group did not overlap. With these four tag groups, cascading style annotation could be applied [31]. To automate the annotation process, a biomedical entity extraction method using the supervised machine learning algorithm, support vector machines (SVM), was applied to their literature library. Supervised machine learning algorithms learn patterns and make predictions based on a set of training data. The training data for this system was generated by first parsing the text using a part-of-speech (POS) tagger, with tag category and boundary represented using the BIO format. The part-of-speech information, category, and context surrounding the term where used as features (or parameters) for the machine learning algorithm. For the source material, a publicly available chemical entity recognizer, OSCAR3-a5, was first used to parse the papers. However, since the precision (the percentage of correctly identified entities over all the entities identified by the system) of OSCAR-a5 was poor (0.59), the group developed a custom chemical entity recognizer called CNER, where they improved issues related to chemical symbol and acronym confusion. CNER had improved precision (0.92) with similar recall (0.97 compared to 0.99 for OSCAR-a5). Recall is the percentage of correctly identified entities over all the entities in the datatset. The authors also used a text chunk annotator based on the sequence labeling tool called YamCha (available at http://chasen.org/~taku/software/yamcha/) and a POS tagger called GPoSTTL (available at http://gposttl.sourceforge.net/).

The tool was further improved by applying a physical quantities list (based on the one listed on the website chemistry.about.com) to refine the extraction of two tags: evaluation parameter and experiment parameter [31]. However, their annotated library only expanded from two to five papers, and the group only used two papers to test their improved system. The group also further improved their CNER, renaming it SERB-CNER or syntactically enhanced rule-based chemical entity recognizer. SERB-CNER still focused on the Source Material tag. Here the POS tagger used was rb tagger. The machine learning system used was CRF++. This new system had recall improvements of 4–7% depending on which parameter was examined.

### Nanomedicine

Through targeted and activatible delivery, nanomedicine has the potential to greatly improve drug efficacy while reducing side effects. Improved design can also address emerging challenges to disease treatment such as adaptive resistance. Despite the promise, few nanomedicines have successfully advanced from the bench to the clinic. For both developing and marketed nanomedicines, there still remain questions on the long-term safety. Two groups have developed NLP-based systems to annotate and classify nanomedicine articles or clinical trials.

#### Nanotoxicity Searcher

The Nanotoxicity Searcher is a tool to automatically annotate nanomedicine and nanotoxicology literature using pattern matching techniques [9,16,33]. The group used ABNER (available at http://pages.cs.wisc.edu/~bsettles/abner/), a biomedical named entity recognizer, to identify names of nanomaterials (NANO), potential routes of exposure (EXPO), target organs and/or organisms (TARGET), and types of toxicity/damage (TOXIC) [16,34]. ABNER contains the supervised machine learning algorithm linear-chain conditional random fields (CRFs) from Mallet (available at http://mallet.cs.umass.edu/), an open source freely available Java-based statistical natural language processing toolkit [35]. To create training data for the CRF, the authors manually annotated 300 sentences collected from 654 abstracts retrieved in PubMed after searching “nanoparticles/toxicity (MeSH major topic)”. For example, the authors manually labeled the sentence

“The purpose of this study was to review published dose-response data on acute lung inflammation in rats after instillation of titanium dioxide particles or six types of carbon nanoparticles.”

with the NANO, EXPO, TARGET and TOXIC mentions within the sentence

“*The purpose of this study was to review published dose-response data on acute <TARGET> ****lung**** </TARGET> <TOXIC> ****inflammation**** </TOXIC> in <TARGET> ****rats**** </TARGET> after <EXPO> ****installation ****</EXPO> of <NANO> ***titanium dioxide particles** </NANO> or six types of <NANO> **carbon nanoparticles** </NANO>).”

Features extracted from the context surrounding the mentions were used to train the CRF.

The performance of their NER software was measured based on three factors: precision, recall, and F-measure score. F-measure is the harmonic mean of precision and recall. The authors evaluated how well their system performed in identifying the entire entity string (entity-level) and partial matches (token-level). For each level, their results were reported to be greater than 0.85, with almost all factors examined at the token level greater than 0.9. The performance of the Nanotoxicity Searcher was also compared to a baseline method, which combines a dictionary-based approach with a term selection scheme. The dictionary was created manually from the same 300 sentences used to train the CRF plus terms identified from two ontologies, the Foundational Model of Anatomy (FMA) and the NanoParticle Ontology [36]. The results demonstrated that overall the CRF method obtained a significantly higher F-measure than the baseline.

#### NanoSifter

The NanoSifter, which focused on a specific type of ENM, is finer grained than the Nanotoxicity Searcher, which used four broad nano entities encompassing all types of ENMs [15]. NanoSifter was designed to identify quantitative data (i.e., numerical values for different characterization parameters) associated with a specific class of dendrimer, poly(amidoamine) (PAMAM), which shows promise for cancer treatment. PAMAM dendrimers are three-dimensional, highly-branched, polymeric ENMs synthesized by growing shells of branched molecules from a central core ethylenediamine molecule. Each doubling of the number of amine surface groups constitutes a new shell or generation.

The NanoSifter algorithm contains two steps. The first to identify possible mentions of the entities associated with PAMAM, and the second to associate the numeric values and dendrimer property terms. The entities associated with PAMAM were based on the NanoParticle Ontology and included: (1) hydrodynamic diameter, (2) particle diameter, (3) molecular weight, (4) zeta potential, (5) cytotoxicity, (6) IC_50_, (7) cell viability, (8) encapsulation efficiency, (9) loading efficiency, and (10) transfection efficiency [17]. To identify mentions associated with PAMAM entities, the authors utilize the freely available open source NLP pipeline General Architecture for Text Engineering (GATE, https://gate.ac.uk/) and its IE module ANNIE (a Nearly-New Information Extraction System, https://gate.ac.uk/ie/annie.html) [37]. GATE, originally developed by the University of Sheffield, is a widely employed suite of Java tools developed for the processing unstructured text [37]. ANNIE is an information extraction module within GATE that contains a tokenizer, sentence splitter, part-of-speech tagger and named entity extractor. The named entity extractor of ANNIE is tailored to extract entities such as persons, organizations and dates, but the components are highly configurable and can be adapted to extract a variety of entities.

To create a training set for the entity extractor, two domain experts annotated 100 articles for the numeric values and dendrimer property terms using the Java Annotations Patterns Engine (JAPE) and integrating components from ANNIE. The training data was then utilized by ANNIE’s IE module to identify mentions associated with PAMAM. The identified numerical values cannot be automatically assumed to associate with a PAMAM property. Therefore, to determine if the associated numeric values of the PAMAM entities were referring to the dendrimer property, the authors utilized a proximity metric. The proximity metric requires the mention of a PAMAM property to be within so many characters of the property term. This provides the system with context information used in the literature when referring to the entity. The authors selected a proximity distance metric threshold of 200 characters based on preliminary experiments using the training set. Too large of a proximity metric provides the system with too much information to accurately discriminate whether the word is an entity, which increases the false positive rate, whereas too little of a proximity metric does not provide the system with enough context information. Evaluating their results using precision, recall and F-measure metrics showed that their algorithm obtained a high accuracy and recall when identifying entities associated with the PAMAM properties. The performance of NanoSifter was based on comparison with annotations generated by researchers working in the Ghandehari lab at the University of Utah. Overall, NanoSifter demonstrated good recall (95–100% - 99%), poor precision (59–100% - 84%), a passing F-measure (73–100% - 91%).

#### Clinical trial document classifier

De la Iglesia et al. proposed a method to automatically classify clinical trial summaries as those testing nanotechnology products and those testing conventional drugs [38]. A benefit of this system is that it can automatically identify summaries of interest for further processing by more computationally intensive systems such as those discussed elsewhere in this review. Looking for just the term “nano” is not sufficient to determine if a summary contains nanotechnology products because many summaries do not explicitly state that they are testing nanotechnology products. For example, many nanotechnology products encapsulate insoluble or highly cytotoxic drugs within liposomal or micellar particles, which alters the kinetics of the drug in the body.

To develop their system, the group used the Natural Language Toolkit (NLTK, http://www.nltk.org/), a suite of freely available, open source, Python-based modules developed for processing unstructured text. They evaluated seven supervised machine learning algorithms implemented in the package: (1) multinomial naive Bayes classifier, (2) decision trees, (3) stochastic gradient descent (SGD) logistic regression, (4) L-1 regularized logistic regression, (5) L-2 regularized logistic regression, (6) linear support vector machine and (7) polynomial support vector machine. The authors explored four vector-based methods for representing the document each using a “bag-of-words” approach containing unigrams (single content words) and bigrams (sequence of two content words) as features (or parameters) for the machine learning algorithm. The first is a binary representation, where a zero or one is used to indicate the absence or presence of the feature in the summary. The second is a feature-based representation, which uses the number of times the feature occurred in the summary. The third is inverse-document frequency (IDF), which quantifies how discriminative a feature is based on the number of documents it occurred within. And lastly, the fourth is term frequency-inverse document frequency (TFIDF), which weights IDF based on how often the term occurs.

The authors trained their algorithm on 1000 clinical trial summaries from clinicaltrials.gov, where 500 were nanomedicine-focused (nano) and 500 were not involving any nanomedicines or nanodevices (non-nano). The author evaluated their system using the leave-one-out and 10-fold cross validation evaluation methodology and report the overall: (1) precision, (2) recall, (3) F-measure, (3) true-positive vs false-positive rates, (4) Mathews correlation coefficient (MCC) and (5) area under the curve (AUC). The MCC measures the quality of the nano/non-nano classification by the system and the AUC measures the discriminativeness of the classifier. The results show an F-measure greater than 0.85 regardless of the machine learning algorithm or feature representation. The overall results indicate that the context within the unigram and bigram features is able to discriminate between non-nano and nano clinical summaries.

The authors describe several advantages of automatically categorizing clinical trials investigating nano versus non-nano drugs. These include facilitating comparisons between clinical trials testing nano and non-nano drug formulations involving the same active ingredient (e.g., Doxil = pegylated liposome [nano] encapsulated *doxorubicin* compared to Adriamycin = *doxorubicin*). In addition, categorization could facilitate information retrieval by users interested in this distinction. In the consumer product arena, labeling consumer products containing ENMs has been discussed widely, and a similar NLP categorization tool tailored to consumer products could potentially facilitate the categorization of products containing nanomaterials or generated using nanotechnology-based processes from those not involving nanotechnology.

### Environmental risk assessment

Environmental release and exposure to ENMs is already occurring, and it is the obligation of nanotechnology researchers to also consider the potential effects of commercialized ENMs on human health and environment. A wealth of data has been collected through large-scale centers, which in the U.S. include the Center for Biological and Environmental Nanotechnology (CBEN) and the two Centers for Environmental Implications of Nanotechnology (CEIN and CEINT). Surprisingly, only one group was found to describe the use of NLP techniques in a tool analyzing the environmental nanotechnology literature.

#### NEIMiner

The Nanomaterial Environmental Impact data Miner, or NEIMiner, is a web-based tool built using CMS and Drupal [39]. NEIMiner consists of four parts: 1) nanomaterial environmental impact (NEI) modeling framework – similar to Framework for Risk Analysis of Multi-Media Environmental Systems (FRAMES), 2) data integration, 3) data management and access, and 4) model building. This web-based tool is supported by the company’s previously developed tool, ABMiner (available at http://discover.nci.nih.gov/abminer/). Three databases (ICON, caNanoLab, and NBI) were used as the data sources. Data extraction was performed using application programming interface (API) calling via web services and data scraping via parsing web pages. The model building component of NEIMiner utilizes machine learning algorithms from ABMiner, such as nearest neighbor algorithms, tree algorithms and support vector machines. This allows for the systematic evaluation of a variety of algorithms. The model building component also contains a meta-optimizer, which automatically iterates through the algorithms in ABMiner that can be used to solve the input problem to determine which algorithm will provide the most optimal results. To demonstrate the applicability of the model building component, the authors developed a predictive model based on the Nanomaterial-Biological Interactions (NBI) knowledge base. The NBI includes data on the mortality, delayed development and morphological malformations of embryonic zebrafish due to the toxicity of various nanomaterials including metal nanoparticles, dendrimer, metal oxide and polymeric materials [40]. Java Applets were used to visualize the data in 3D histograms and scatterplots. NEIMiner was hosted at http://neiminer.i-a-i.com but is no longer accessible.

## Conclusion

### NLP perspective

Nine nanoinformatics systems utilizing NLP have been described in the literature. Table 1 shows the components of these systems from a NLP perspective. “NLP tasks” describes the applications discussed by the researchers when developing their system. “NLP subtasks” shows the underlying NLP components that were utilized within the systems. For example, NanoMapper, a patent analyzer developed by Li et al., utilized a part-of-speech (POS) tagger and parser within their system to automatically annotate the words in the document with their part-of-speech and extract the phrasal chunks from the sentences [25]. Similarly, the TechPerceptor system developed by Yoon et al. utilizes a stemmer in order to normalize words to their base form, and sentence similarity algorithms to compare how close the contextual content of one sentence is with another [26].

**Table 1:** Nanoinformatic system components from an NLP perspective.

		Nano Porter	Nano Mapper	Tech Perceptor	Text Mining Framework	Nano Device F & C	Nano Toxicity Searcher	Nano Sifter	Clinical Trial Doc. Class.	NEI Miner

machine learning algorithm	CRF					×	×			
	decision trees								×	×
	logistic regression								×	
	naive Bayes								×	
	nearest neighbor									×
	SVM					×			×	×

algorithm class	machine learning						×		×	×
	pattern matching							×		
	clustering	×	×	×	×					

visualization	visualization modules	×	×	×	×					×

taxonomy	FMA (in UMLS)						×			
	MeSH (in UMLS)						×			
	WordNet			×						
	NanoParticle Ontology						×			

NLP tools	GATE ( NLP Toolkit)							×		
	Xconc Suite (annotator)					×				
	ABMiner (NLP Toolkit)									×
	Abner (NER)						×			
	YamCha (Parser)					×				
	GPoSSTTL (POS Tagger)					×				
	ANNIE (GATE module)							×		
	Mallet (NLP Toolkit)						×			
	NLTK (NLP Toolkit)								×	

NLP sub task	POS tagging	×	×	×		×				
	parsing	×	×			×				
	concept mapping			×						
	stemming			×						
	sentence similarity			×						

NLP task	document classification								×	
	document clustering	×								
	entity extraction					×	×	×		×
	information retrieval	×				×				
	patent analyzer	×	×	×	×					
	summarization	×								
	topic identification	×	×							

Many of the nanoinformatics systems were implemented using pre-existing NLP software packages. These NLP packages were developed to perform specific tasks, such as Abner, a biomedical named entity extractor, or more general NLP systems that provide various NLP tools such as Mallet and Natural Language Toolkit (NLTK) [34–35]. Utilizing and adapting these previously developed NLP tools allows for nanoinformatics researchers to build their automated systems without needing to develop low level NLP functionality. There were three main types of algorithms utilized by the systems: machine learning, pattern matching and clustering. The most common was machine learning algorithms such as Conditional Random Fields and Support Vector Machines (SVMs). These algorithms require manually annotated training data. For example, in building the Nanotoxicity Searcher, Garcia-Remesal et al. manually annotated documents for various nanoparticles and their toxicological hazards to train their entity extraction system [16]. In many cases, the annotation toolkit (if used) was not reported, but two annotation systems were mentioned in the articles reviewed: 1) GATE and 2) XConc Suite.

Lastly, although not specifically an NLP component, five groups incorporated visualization of the extracted information as part of their system. Visualization provides researchers with additional capabilities to explore and analyze the data.

### Data perspective

Table 2 shows the components of the nanoinformatics systems from a data perspective. With the growing number of nanotechnology publications, more refined databases that automatically identify records (e.g., articles, patents, grants, clinical trials) relevant to specific ENMs or properties can greatly facilitate trend analyses. The amount of information gathered automatically differed widely between the systems reviewed. The Clinical Trial Document Classifier focused on differentiating between two variables, nanotechnology products and non-nanotechnology products [38]. The four patent mining systems (i.e., NanoPort, NanoMapper, TechPerceptor, and Text Mining Framework) primarily extracted publication information, which allowed for patents to be clustered by date, inventor, country, and institution. However, the TechPerceptor also extracted information on nanomaterial type and synthesis method [26]. Moving beyond bibliographic information, the Nanodevice Fabrication and Characterization Analyzer automatically extracted nanodevice physico-chemical characterization properties as well as the fabrication and evaluation parameters and their associated values [30]. Comparing the parameters that were extracted to the proposed minimum information for nanomaterials characterization, referred to as MINChar in the table, 64% of parameters were captured [41]. This system was trained using two annotated articles, and its application to a larger literature corpus has not been published. This may be due to future plans to integrate a system, similar to the patent analyzers, where the extracted data are associated with the citation information.

**Table 2:** Nanoinformatic system components from a data perspective.

		**MIN Char**	Nano Porter	Nano Mapper	Tech Perceptor	Text Mining Framework	Nano Device F & C	Nano Toxicity Searcher	Nano Sifter	Clinical Trial Doc. Class.	NEI Miner

publication information	citation (e.g., author, journal, date)		×	×	×	×		×			×
	laboratory/ organization			×		×					
	location		×	×		×					
	content description		×	×	×						
	patent classification (e.g., US, EU)		×	×	×						

physico- chemical character- ization	particle diameter	**×**					×		×		×
	particle size distribution	**×**									×
	hydrodynamic diameter								×		
	agglomeration and/or aggregation	**×**									×
	shape	**×**					×				×
	core composition	**×**			×		×	×			×
	crystallinity/crystalline state	**×**					×				×
	surface area	**×**					×				
	surface charge/zeta potential	**×**					×		×		×
	surface chemistry	**×**					×				×
	purity	**×**					×				×
	stability	**×**									
	solubility	**×**									
	concentration (mass, number, SA)	**×**									×
	method of synthesis/preparation	**×**			×		×				×
	molecular weight								×		

exposure	exposure media										×
	exposure pathway/route							×			×
	exposure duration										×
	exposure dose										×

biological response	bioavailability/uptake										×
	biomagnification										×
	cell viability							×	×		
	cytotoxicity							×	×		×
	inflammatory response							×			
	genotoxicity							×			×
	EC_50_ (ppm)							×			
	IC_50_							×	×		
	LC_50_ (ppm)							×			
	organ response							×			
	whole organism response							×			×

The amount of physico-chemical characterization data extracted by the systems analyzing literature for exposure and biological response data (i.e., Nanotoxicity Searcher, NanoSifter, and NEIMiner) varied greatly. Focused primarily on the toxicity endpoints, the Nanotoxicity Searcher extracted several biological response endpoints but only associated these effects with the ENMs’ core composition [16]. The NanoSifter collected size, surface charge and molecular weight data beyond the core composition, which was fixed to PAMAM [15]. Incorporating almost 80% of the minimum characterization data, the NEIMiner appears to be the most comprehensive with regards to extraction of physico-chemical characterization properties.

When assessing the human health or environmental impact of ENMs, it is important to recognize that risk is a function of exposure and hazard. Without exposure, there is no risk. All substances are potentially hazardous depending on the dose or concentration encountered. In addition, the biological response data of interest can be dependent upon the application. Nanomedicine applications are often evaluated using performance parameters, such as drug loading efficiency and efficacy, in addition to biological response, such as cytotoxicity or IC_50_. Since efficacy and cytotoxicity are dependent upon the administered dose, concentration and exposure dose parameters are critical for the interpretation of this data. While text mining is useful, it is only the first step. Current nano-focused NLP systems are not sufficient to reveal relationships or connections between data. Close collaboration and communication between nanotoxicology and nanoinformatics researchers will provide interpretive context so that computer understandable patterns can be developed to enable future knowledge discovery from the literature.

### Recommendations

There is a critical need to automatically extract and synthesize knowledge and trends from nanotechnology literature. New ENMs are continuously being discovered and NLP approaches can semi-automate the cataloguing of ENMs and their unique physico-chemical properties. As shown in this review, various NLP methods have been used for patent mining, nanomaterial/device characterization, nanomedicine, and environmental risk assessment. We believe these approaches can be expanded upon to automatically aggregate studies on the exposure and hazard of ENMs as well as link the physico-chemical properties to the measured effects. Towards this end, we conclude with the following recommendations:

- Add the NPO to the Unified Medical Language System (UMLS). → Impact: provide a nano-specific terminology source that can be used by pre-existing systems that currently utilize sources from the UMLS.
- Create a publicly available annotated corpus for nanotechnology. → Impact: develop new nanoinformatics tools; provide a benchmark dataset to compare nanoinformatic systems.
- Encourage authors to include more experimental details, such as the minimum characterization data, in their manuscripts. → Impact: increase experimental reproducibility and inter-study comparison.
- Encourage researchers to add nanoinformatics tools to freely available, online repositories, such as nanoHUB or NCIPhub. → Impact: Promote broader participation in the nanoinformatics field.
